# Adaptive Feature Extraction Using Sparrow Search Algorithm-Variational Mode Decomposition for Low-Speed Bearing Fault Diagnosis

**DOI:** 10.3390/s24216801

**Published:** 2024-10-23

**Authors:** Bing Wang, Haihong Tang, Xiaojia Zu, Peng Chen

**Affiliations:** 1 School of Marine Engineering Equipment, Zhejiang Ocean University, Zhoushan 316022, China; wb18354889982@163.com (B.W.); home8882022@126.com (X.Z.); 2 Graduate School and Faculty of Bioresources, Mie University, Tus 514-8507, Mie, Japan; chen@bio.mie-u.ac.jp

**Keywords:** fault diagnosis, VMD, sparrow search algorithm, kurtosis criterion, signal analysis, low-speed bearing

## Abstract

To address the challenge of extracting effective fault features at low speeds, where fault information is weak and heavily influenced by environmental noise, a parameter-adaptive variational mode decomposition (VMD) method is proposed. This method aims to overcome the limitations of traditional VMD, which relies on manually set parameters. The sparrow search algorithm is used to calculate the fitness function based on mean envelope entropy, enabling the adaptive determination of the number of mode decompositions and the penalty factor in VMD. Afterward, the optimised parameters are used to enhance traditional VMD, enabling the decomposition of the raw signal to obtain intrinsic mode function components. The kurtosis criterion is then used to select relevant intrinsic mode functions for signal reconstruction. Finally, envelope analysis is applied to the reconstructed signal, and the results reveal the relationship between fault characteristic frequencies and their harmonics. The experimental results demonstrate that compared with other advanced methods, the proposed approach effectively reduces noise interference and extracts fault features for diagnosing low-speed bearing faults.

## 1. Introduction

Bearings are critical components in rotating machinery, and their condition directly influences the performance of the entire mechanical system, particularly in low-speed machinery [1]. Bearing failure can disrupt production processes, leading to economic losses and potentially catastrophic accidents. Therefore, timely and accurate detection of bearing faults is essential for ensuring the reliability and stability of low-speed rotating machinery [2,3].

When a bearing malfunctions during operation, it generates abnormal vibration signals due to mutual impacts [4,5]. With advancements in sensor technology, vibration signal processing-based methods for bearing diagnosis have continuously evolved and are now among the most widely used techniques for bearing fault detection globally [6,7]. Several modern signal processing technologies are currently applied in bearing fault diagnosis, including empirical mode decomposition (EMD), ensemble empirical mode decomposition (EEMD), and local mean decomposition (LMD). EMD, in particular, is an effective method for adaptively and unsupervisedly extracting fault features from nonlinear and non-stationary vibration signals, which are decomposed into intrinsic mode functions (IMFs). However, EMD has several drawbacks, including end effects and mode mixing [8]. EEMD effectively reduces mode mixing in EMD, while LMD addresses the end effects encountered in processing non-stationary signals by correcting over-enveloping and under-enveloping [9]. Despite these improvements, mode mixing remains a significant issue in fault diagnosis and requires careful attention.

To better extract fault features and obtain fault information, variational mode decomposition (VMD) has been proposed to address the abovementioned issues. VMD determines the centre frequency and bandwidth of each IMF by adaptively identifying the number of IMFs [10]. Owing to its strong capability to suppress mode mixing, VMD is widely used in bearing fault diagnosis [11]. Wang employed VMD to detect multiple rubbing-induced signatures for rotor–stator fault diagnosis using both numerically simulated response signals and practical vibration signals [12]. He proposed an improved grey wolf optimiser (IGWO), incorporating various strategies to enhance VMD performance in processing the early weak signals of train bearings [13]. Li introduced an independent-oriented VMD method based on correlation analysis to adaptively extract weak and compound fault features of wheel-set bearings [14].

However, the effectiveness of VMD is highly influenced by the number of IMFs and the penalty factor, which are typically determined based on manual experience. In addition, VMD has difficulties in effectively extracting fault signals when dealing with complex signal processing with strong background noise interference. Therefore, setting appropriate parameters is crucial for optimising VMD performance. To address this, Li [15] combined the improved particle swarm optimisation (PSO) algorithm with enhanced envelope entropy to optimise the VMD layer and penalty parameters. Yan [16] proposed a novel robust intelligent fault diagnosis method for rolling bearings, based on sparsity-assisted, parameter-adjustable VMD, to detect fault information under harsh operational conditions where signals are easily obscured by strong noise interference. Wang [17] proposed an adaptive extraction algorithm (OEGOA-VMD) by introducing a new exponentially optimised mean characteristic energy ratio (OCFER) as the fitness function of OEGOA to determine the optimal parameters for VMD. In another study, Wang [18] developed a parameter-adaptive VMD method using a future search algorithm based on sine cosine algorithm-variational mode decomposition (FSASCA-VMD) combined with Graph Sample and Aggregate-self attention (GraphSAGE-SA). This approach estimates the number of IMF decompositions and the penalty factor to match the analysed vibration signal. Wu [19] introduced a PSO algorithm, which calculates the fitness function based on fuzzy entropy, to adaptively search for the optimal mode number and penalty factor in VMD, improving the accuracy of sequence decomposition and forecasting.

Most of the methods proposed by the aforementioned scholars have effectively addressed bearing fault diagnosis in medium- to high-speed conditions. However, feasible solutions for machines operating at low speeds (defined as 20–600 rpm) [20] remain limited. There is an urgent need for a high-precision fault diagnosis method capable of extracting fault information submerged in noise and other interference components under low-speed conditions. To address these challenges, a new parameter-adaptive VMD method based on the SSA is proposed for bearing fault diagnosis, using the minimum mean envelope entropy (MEE) as the fitness function to optimise VMD parameters. The selected IMFs, based on the kurtosis criterion, were then reconstructed for envelope analysis to extract the fault feature frequency of bearings. The results demonstrate that the proposed method outperforms EMD and VMD in terms of decomposition results and fault feature extraction for low-speed bearing fault diagnosis. The main contributions of this paper are as follows:

(i) The proposed method addresses the limitations of manually selecting VMD parameters through empirical methods and trial-and-error, which are commonly used in VMD. By integrating the SSA into the VMD decomposition and utilising the MEE as the adaptive function for the SSA to select the optimal VMD parameters, the efficiency of VMD decomposition is significantly improved.
(ii) To mitigate interference from noise components, IMF components from VMD decomposition are classified into useful and noise components using the kurtosis criterion. The useful IMF components are then reconstructed to obtain a denoised signal.
(iii) The proposed SSA-VMD method addresses the challenge of extracting fault characteristics from low-speed bearings, even in the presence of strong environmental noise.

The remainder of this study is organised as follows:

Section 2 outlines the principles and process of parameter adaptation in VMD, including the theory of VMD and the principles of the SSA. Section 3 details the complete fault diagnosis process using SSA-VMD and introduces the kurtosis criterion for signal reconstruction. Section 4 evaluates the effectiveness of the SSA-VMD method combined with the kurtosis criterion through simulation signals. Section 5 assesses the method’s effectiveness using experimental results from actual vibration signals. Section 6 provides the conclusions of this study.

## 2. Basic Theory

### 2.1. Variational Mode Decomposition

VMD is an adaptive signal decomposition algorithm that builds on classical Wiener filtering and the Hilbert–Huang transform [21,22].

The constrained variational problem can be formulated as follows:

(1) $$\begin{array}{l} \min_{\left\{uk\},\{wk\right\}},\left\{{\sum_{k}\left\|\partial_{t}\left[\left(\delta (t)+\frac{j}{\pi t}\right)*u_{k}(t)\right]e^{-jw_{k}t}\right\|}_{2}^{2}\right\}\\ \sum_{k}u_{k}=f \end{array}$$

where $u_{k}(t)$ represents the *k* component of the original signal after VMD; *t* denotes time; f(t) is the original signal; $\omega_{k}$ stands for the central frequency of each mode component; and * represents the convolution operator.

Equation (2) represents incorporating the augmented Lagrangian function.

(2) $$L\left(\left\{u_{k}\right\},\left\{w_{k}\right\},\lambda \right)=\alpha {\sum_{k}\left\|\partial (t)\left[\left(\delta (t)+\frac{j}{\pi t}\right)u_{k}(t)\right]e^{-jw_{k}t}\right\|}_{2}^{2}+{\left\|f(t)-\sum_{k}u_{k}(t)\right\|}_{2}^{2}+\langle \lambda (t),f(t)-\sum_{k}u_{k}(t)\rangle$$

where *α* is the secondary penalty factor and λ is the Lagrange multiplier.

The optimal solution of the constrained variational model is shown in Equation (3)

(3) $$u^{n+1}=\underset{u_{k}\in X}{\arg\min}\left\{\alpha {\left\|\partial_{t}\left[\left(\delta (t)+\frac{j}{\pi t}\right)*u_{k}(t)\right]e^{-j\omega_{k}t}\right\|}_{2}^{2}+{\left\|f(t)-\sum_{i}u_{i}(t)+\frac{\lambda (t)}{2}\right\|}_{2}^{2}\right\}$$

where $\omega_{k}$ is equivalent to $\omega_{k}^{n+1}$ and $\sum_{i}u_{i}(t)$ is equivalent to $\sum_{i\neq k}u_{i}{\left(t\right)}^{n+1}$.

Solving Equation (3) in the frequency domain yields the updated expressions for the various mode components.

(4) $${\hat{u}}_{k}^{n+1}(\omega )=\frac{\hat{f}(\omega )-\sum_{i\neq k}{\hat{u}}_{k}(\omega )+\frac{\hat{\lambda }(\omega )}{2}}{1+2\alpha {\left(\omega -\omega_{k}\right)}^{2}}$$

Similarly, solving for the central frequency in the frequency domain provides the updated expression $\omega_{k}$.

(5) $$\omega_{k}^{n+1}=\frac{\int_{0}^{\infty }\omega {\left|{\hat{u}}_{k}(\omega )\right|}^{2}d\omega }{\int_{0}^{\infty }{\left|{\hat{u}}_{k}(\omega )\right|}^{2}d\omega }$$

where ${\hat{u}}_{k}^{n+1}(\omega )$ is equivalent to the current residual $\hat{f}(\omega )-\sum_{i\neq k}{\hat{u}}_{i}(\omega )$ as a Wiener filter; $\omega_{k}^{n+1}$ represents the centre of the current modal function’s power spectrum. Performing an inverse Fourier transform $\left\{{\hat{u}}_{k}(\omega )\right\}$ on yields $\left\{u_{k}(t)\right\}$.

**Figure 1:**
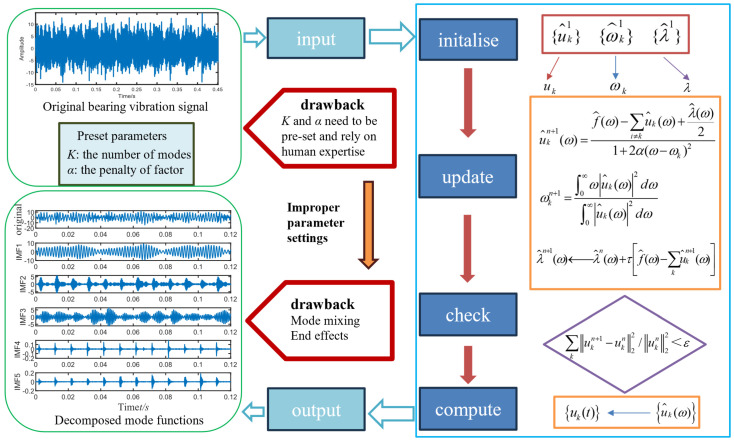
Flowchart of VMD.

The issues with VMD are as follows: the number of modes *k* and the penalty factor *α* must be pre-set, relying on human expertise during the VMD process. The process of VMD is illustrated in Figure 1.

### 2.2. Sparrow Search Algorithm

Compared with the WOA algorithm and the PSO algorithm, the SSA demonstrates both robustness and fast convergence [23]. For simplicity, Liu [24] categorised the entire sparrow population into producer, scrounger, and early warning groups, establishing corresponding rules to develop a mathematical model.

The position of the sparrows can be represented in the following matrix:

(6) $$X=\left[\begin{array}{cccc} x_{1,1} & x_{1,2} & \cdots & x_{1,d}\\ x_{2,1} & x_{2,2} & \cdots & x_{2,d}\\ \vdots & \vdots & \vdots & \vdots \\ x_{n,1} & x_{n,2} & \cdots & x_{n,d} \end{array}\right]$$

where *n* is the number of sparrows and *d* represents the dimension of the variable to be optimised. The fitness values of all sparrows can be represented by the following vector:

(7) $$F_{x}=\left[\begin{array}{c} f\left(\left[\begin{array}{cccc} x_{1,1} & x_{1,2} & \cdots & x_{1,d} \end{array}\right]\right)\\ f\left(\left[\begin{array}{cccc} x_{2,1} & x_{2,2} & \cdots & x_{2,d} \end{array}\right]\right)\\ \vdots \\ f\left(\left[\begin{array}{cccc} x_{n,1} & x_{n,2} & \cdots & x_{n,d} \end{array}\right]\right) \end{array}\right]$$

where *n* is the number of sparrows and *f* represents the fitness value of the individual. During each iteration, the position update expression for the producer is as follows:

(8) $$x_{i,j}^{t+1}=\{\begin{array}{c} x_{i,j}^{t}\cdot \exp(\frac{-i}{\beta \cdot T_{\max}}),R_{2}<f_{sT}\\ x_{i,j}^{t}+Q\cdot L,R_{2}\geq f_{sT} \end{array}$$

where $x_{i,j}^{t}$ represents the value of the j−th dimension of the i−th sparrow at the t iteration; $T_{\max}$ is the maximum number of iterations; β∈(0,1) is a random number; and $f_{ST}\in [0.5,1.0]$ and $R_{2}\in \left[0,1\right]$ represents the alarm value and safety threshold, respectively. *Q* is a random number following a normal distribution, and *L* is an internal matrix with all its elements set to 1. $R_{2}<f_{ST}$ indicates the absence of predators in the vicinity, prompting foragers to switch to an expansive search mode.

The position update formula for the scrounger is as follows:

(9) $$x_{i,j}^{t+1}=\{\begin{array}{c} Q\cdot \exp(\frac{X_{worst}^{t}-X_{i,j}^{t}}{i^{2}}),\;i>n/2\\ X_{p}^{t+1}+\left|X_{i,j}^{t}-X_{p}^{t+1}\right|\cdot A^{+}\cdot L,\;otherwise \end{array}$$

where $x_{worst}$ is the worst position on the board and is the optimal position of the producer. *A* is a 1 × d-dimensional matrix with its elements randomly assigned as −1 or 1. Furthermore, $A^{+}=A^{T}{\left(AA^{T}\right)}^{-1}$. *i* > *n*/2 individuals with lower adaptability are more likely to face starvation and need to fly elsewhere to search for food.

The position update formula for the early warning is

(10) $$x_{i,j}^{t+1}=\{\begin{array}{c} X_{best}^{t}+\beta \cdot \left|X_{i,j}^{t}-X_{best}^{t}\right|,\;f_{i}>f_{g}\\ X_{i,j}^{t}+K\cdot \left(\frac{\left|X_{i,j}^{t}-X_{worst}^{t}\right|}{\left(f_{i}-f_{w}\right)+\varepsilon }\right),\;f_{i}=f_{g} \end{array}$$

where $x_{best}$ represents the current best position. β is a random number with a mean of 1 and a variance of 0, following a normal distribution. A∈[0,1] is a random number. $f_{g}$ represents the best fitness among sparrows in the current global situation, $f_{i}$ denotes the fitness of sparrows in the current situation, and $f_{\omega }$ represents the worst fitness. ε is the smallest constant. In simpler terms, $f_{i}>f_{g}$ indicates that the sparrow is at the edge of the flock.

The detailed steps of the SSA are provided in Algorithm 1.
**Algorithm 1: Framework of the SSA****Input:** *T*_max_: the maximum iterations; *PD*: the number of producers; *SD*: the number of sparrows who perceive the danger; *R*_2_: the alarm value; *N*: total number of sparrows**Output:** *X_best_*: the current global optimal location; *f_g_*: fitness (*X_best_*)**while (***t* < *T*_max_**)**
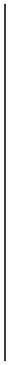
Rank the fitness values and find the current best individual and the current worst individual.
*R*_2_ = rand (1)  # Update the alarm value randomlyfor i=1:PD
 Using Equation (8), update the sparrow’s location
endfor i=(PD+1):N
 Using Equation (9), update the sparrow’s location
endfor i=1:SD

 Using Equation (10), update the sparrow’s location
end forGet the current new location
If the new location is better than before, update it
*t* = *t* + 1**end while****Return ***X_best_*, *f_g_*

### 2.3. Kurtosis Criterion

The VMD algorithm can decompose signals into a finite set of IMFs based on their inherent characteristics [25,26]. However, the IMFs obtained through VMD have the following drawbacks: (i) VMD can result in some IMFs being contaminated with significant amounts of noise, and (ii) some IMFs may lose crucial information that accurately reflects the signal characteristics [27]. Often, the IMFs obtained through the SSA-VMD algorithm decompose into signals that are mostly noise or interference, with only a few components accurately reflecting bearing fault characteristics. Therefore, it is essential to filter out the effective IMFs with richer fault information based on certain selection criteria to achieve noise reduction and enhance fault feature extraction.

Common methods for selecting the optimal IMF component include (i) the kurtosis criterion and (ii) the correlation coefficient [28,29]. While the correlation coefficient method effectively detects signal similarity and linear relationships, the kurtosis criterion provides the following advantages for fault diagnosis: (i) high sensitivity to shock and spike signals; (ii) strong resistance to noise; and (iii) high computational efficiency. These characteristics make the kurtosis criterion particularly effective for high-order feature extraction and nonlinear signal processing.

Kurtosis is a dimensionless parameter that characterises the peak of a waveform [30]. It is mathematically defined as the kurtosis value *K*, which is given by

(11) $$K=\frac{E{\left(x-\mu \right)}^{4}}{\sigma^{4}}$$

where E(x) represents the expected value of the variable *x*; μ is the mean value of the signal; and σ is the standard deviation of the signal. A larger kurtosis value indicates a more severe fault [31].

## 3. Proposed Method for Low-Speed Bearing Fault Diagnosis Based on SSA-VMD

VMD is a signal processing technique that decomposes signals into modal components of different frequencies [32]. However, it has the following drawbacks when applied to raw signals: (i) The key parameters *k* and *α* of VMD depend on manual expertise and require careful tuning for optimal decomposition, which can lead to inefficient results. (ii) Extracting fault features is challenging because of the increased signal envelope entropy caused by complex signal components after VMD. This article proposes using mean envelope entropy as the fitness function and optimising the decomposition parameters *k* and α of VMD with the sparrow search algorithm. The SSA-VMD method proposed in this paper offers the following advantages over VMD: (i) The optimal parameters *k* and *α* are adaptively determined through the SSA-VMD approach. (ii) SSA-VMD addresses the challenges of analysing low-speed bearing signals by effectively separating fault signals with strong characteristic energy from the original signals, which are heavily influenced by noise. The MEE is an enhancement of Shannon entropy [33], and it provides a more accurate reflection of the sparsity characteristics of the original signal [34] compared with directly calculating the Shannon entropy of the original signal [35,36]. The expression for *E_p_* is as follows:

(12) $$\begin{array}{l} \begin{array}{c} E_{p}=-\sum_{j=1}^{N}\left(p_{j}\mathrm{lg}p_{j}\right)\\ p_{j}=a(j)/\sum_{j=1}^{N}a(j) \end{array}\\ a_{j}=\sqrt{{\left[S(j)\right]}^{2}+{\left\{H\left[S\left(j\right)\right]\right\}}^{2}} \end{array}$$

where a(j) represents the envelope signal derived from the original signal through Hilbert demodulation analysis. $P_{j}$ is the normalised representation of a(j). *N* denotes the number of envelope signals.

To determine the optimal parameter combination under different operating conditions, the minimum value of the MEE is calculated as the fitness criterion. The fitness function is represented by Equation (13).

(13) $$mean\left\{E_{p}\right\}=mean\left\{E_{p_{1}},E_{p_{2}},\cdots ,E_{p_{k}}\right\}=\frac{1}{k}\sum_{i=1}^{k}E_{p_{i}}$$

where *k* represents the number of IMF components obtained through VMD. As the MEE value decreases, the complexity of IMFs is lower and the signal becomes more stable.

For the proposed SSA-VMD method, in addition to the main parameters *k* and *α* that need to be set, other parameters that indirectly affect the optimisation of VMD by the SSA need to be discussed, especially the number of producers and population size. We will provide a detailed analysis in Section 5.2 and Section 5.3.

**Figure 2:**
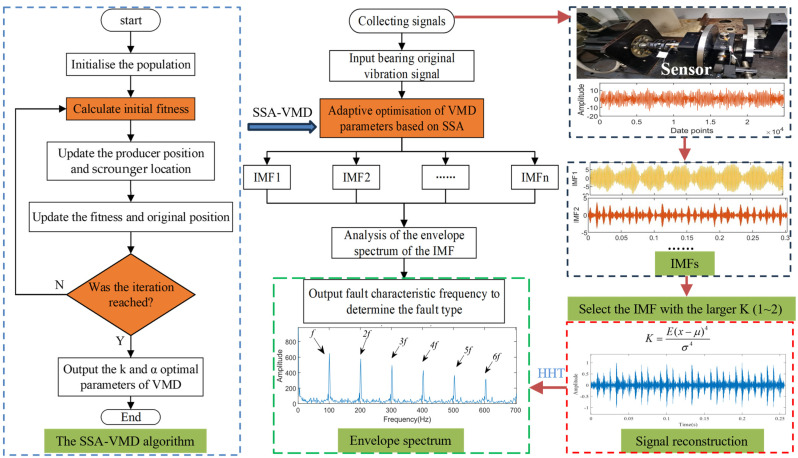
Fault diagnosis flowchart for bearings using parameter-adaptive VMD.

The optimal parameters for VMD can be determined by integrating the optimisation algorithm with the MEE, as outlined in Algorithm 2. In the SSA-VMD process, the minimum MEE is used as the objective function for optimisation. The fault diagnosis process for low-speed bearings based on parameter-optimised VMD is illustrated in Figure 2.
**Algorithm 2: Steps of the implementation of the improved SSA-VMD model****Input**: raw signal**Output**: $\left(k_{best},\alpha_{best}\right)$/* Adaptive optimisation of VMD parameters based on the SSA*/Initialise *I*_max_, *R*_2_, *PD*, *SD*Calculate the initial *MEE*$E_{p}$
Obtain $X_{best}^{T}$
while ($t<T_{\max}$)
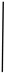
Update the sparrow’s location through Algorithm 1Calculate min $MEE^{T+1}$
If $\min MEE^{T+1}<\min MEE^{T}$, $X_{best}^{T}\leftarrow X_{best}^{T+1}$
end whileobtain ($k_{best}$$,\alpha_{best}$)

## 4. Simulation Signal Analysis and the Performance Testing of Algorithms

### 4.1. Simulation Experiment Based on Low-Speed Bearings

**Figure 3:**
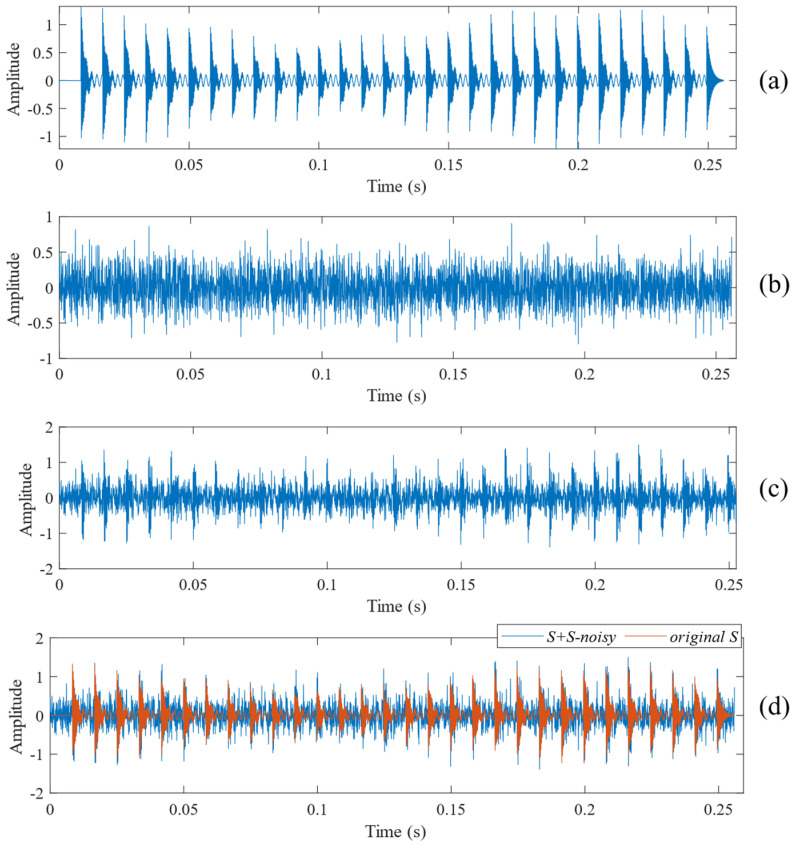
Waveform of the synthesised simulation signal. (**a**) Original vibration signal with bearing fault. (**b**) Gaussian white noise. (**c**) Simulated vibration signal with bearing fault (after adding noise). (**d**) Comparison between raw vibration signal and simulated bearing fault signal.

The simulation signal in Section 4 (as shown in Figure 3) consists of three parts, including the periodic impact component, the vibration component of the bearing fault, and noise. (i) The periodic impact component is part of the background noise, which simulates the periodic vibration generated by the coupling of mechanical components during operation. (ii) The vibration component of the bearing fault is the impact signal generated by mechanical operation collision due to the bearing fault [37]. The purpose of the proposed method is to find this signal from the background noise. (iii) For the noise component, Gaussian white noise (an ideal noise) is introduced in this research. The signal-to-noise ratio of the noise signal is –13 dB, and it is used to quantitatively measure noise.

By accounting for both the measurement system’s uncertainty and adjusting the noise to match the specified SNR, the simulation more accurately mimics practical conditions, allowing for a more meaningful evaluation of the fault diagnosis method. Furthermore, the effectiveness of the proposed method in low-speed bearing fault diagnosis has been preliminarily demonstrated through simulation signals that can quantitatively measure noise and uncertainty.

To further validate the effectiveness of the SSA-VMD method proposed in this study, a fault model is used to simulate the impact signal generated by local defects in the bearing inner ring. The inner ring fault signal is simulated by adding significant white noise. The expression for the simulated signal is as follows:

(14) $$S(t)=S_{impact}(t)+S_{fault}(t)+S_{noise}(t)$$

(15) $$S_{impact}(t)=(1+A_{0}\cos(2\pi f_{r}t))\cdot \exp(-Ct)\cdot \sin(2\pi f_{n}t)$$

(16) $$S_{fault}(t)=A_{fault}\sin(2\pi f_{fault})$$

(17) $$S_{noise}(t)=\sqrt{P_{noise}}\cdot n(t)$$

where $S_{impact}(t)$ represents the periodic impact component. The displacement constant $A_{0}$ is 0.3; the frequency conversion $f_{r}$ is 5 Hz; the attenuation coefficient *C* is 700; and the natural frequency $f_{n}$ is 4 kHz. $f_{fault}$ is the vibration component of bearing failure. The fault vibration frequency is 500 Hz and the fault vibration amplitude $A_{fault}$ is 0.1. $S_{noise}(t)$ represent the Gaussian white noise component. The signal-to-noise ratio of the noisy signal is −13 dB. $P_{noise}$ is the power of noise and n(t) is the Gaussian white noise with a mean of 0 and a variance of 1. The inner circle fault feature frequency is 120 Hz; the sampling frequency is 16 kHz; and the number of analysis points is 4096.

A bearing inner race fault signal with noise was obtained by introducing Gaussian white noise into the original signal, as illustrated in Figure 3. Figure 3a illustrates the relatively clear impact signal, while Figure 3c illustrates that the periodic impact signal becomes less apparent after the addition of Gaussian white noise.

**Figure 4:**
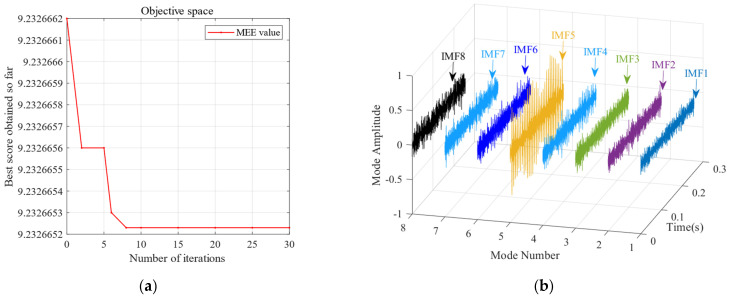
(**a**) VMD parameter optimisation process based on the SSA. (**b**) SSA-VMD decomposition.

**Figure 5:**
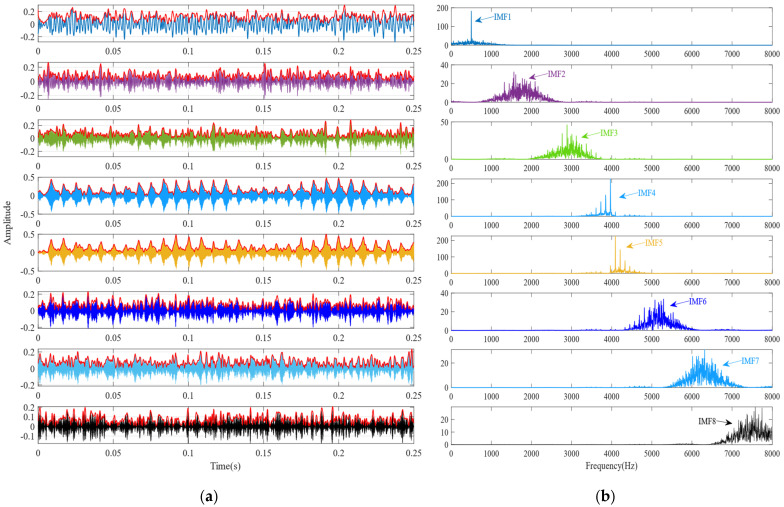
(**a**) Envelope plots of IMF1 through IMF8. (**b**) Frequency domain signals obtained through SSA-VMD decomposition.

**Figure 6:**
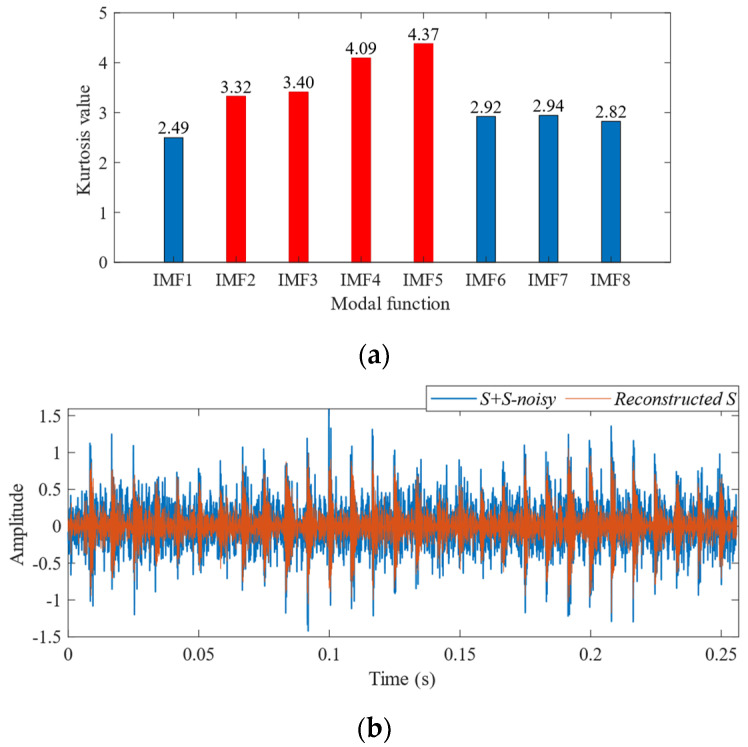
(**a**) Kurtosis values of the IMFs. (**b**) Time-domain diagrams of the reconstructed signals for IMF2–IMF5.

**Figure 7:**
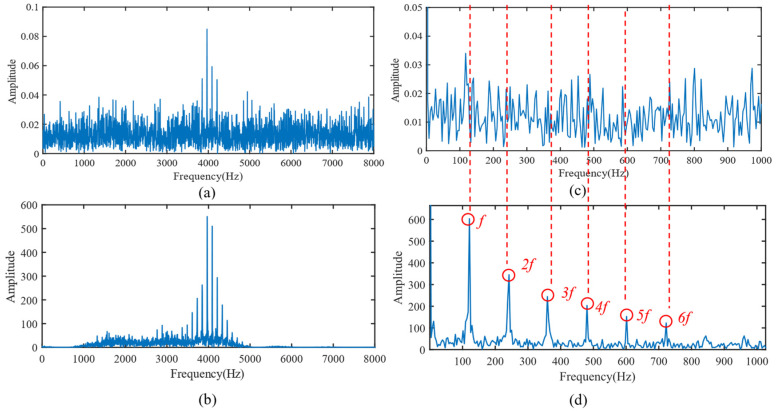
(**a**) Fourier spectrum of the original simulated fault signal. (**b**) Fourier spectrum of the reconstructed signal. (**c**) Envelope spectrum of the original simulated fault signal. (**d**) Envelope spectrum of the reconstructed signal.

To further validate the effectiveness of SSA-VMD, the SSA is first used for adaptive optimisation to determine the number of modes and the penalty factor for VMD under the given operating conditions. The optimisation process, which uses the minimum MEE as the fitness function, is illustrated in Figure 4a. The initial settings for the SSA include the population size, penalty factor, and optimisation bounds for the number of modes, with a maximum iteration count of 30. The vibration signals are then decomposed using SSA-VMD, resulting in optimal parameter combinations of [8, 687] when the minimum MEE reaches 9.2323. Figure 4b presents the IMF1–IMF5 components after VMD decomposition. The envelopes and spectra of IMF1–IMF8 are illustrated in Figure 5a,b. Figure 6a illustrates the kurtosis values for IMF1–IMF5. According to the kurtosis criterion, IMF2–IMF5 are selected for signal reconstruction, as they reveal a more distinct fault impact, as illustrated in Figure 6b. As illustrated in Figure 7a, the frequency domain plot of the noisy simulation signal, obtained after performing a fast Fourier transform, reveals a high amplitude near 4 kHz. Figure 7c illustrates the results of an envelope spectrum analysis on the noisy simulation signal; however, the amplitude corresponding to the specific frequency associated with the bearing fault, as well as its harmonic frequencies, is not significant. The envelope analysis of the reconstructed signal highlights a clear frequency doubling relationship, as illustrated in Figure 7d. This analysis, coupled with the inner ring fault mechanism, confirms the presence of a local inner ring fault in the rolling bearing. The experimental results validate the effectiveness of the proposed method.

### 4.2. Performance Test of the SSA

To thoroughly evaluate the performance of the SSA, unimodal functions, multimodal functions, and fixed-dimensional test functions were chosen to assess the algorithm’s convergence speed, stability, and accuracy. In the experimental environment, the experiments were conducted in Matlab 2022a on a computer with a 3.40 GHz Intel i7 processor and 16 GB of memory. There are six classic test functions to be cited in Table 1.

The maximum number of iterations was set to 1000, and the population size was 100 (n = 100) for each trial. For the whale optimization algorithm (WOA), a population size and maximum iteration equal to 30 and 500 were utilised. The parameters for the grey wolf optimiser (GWO) were configured as follows: the $\vec{a}$ value decreased linearly from 2 to 0, and r1 and r2 ∈ [0, 1]. The parameters of the PSO were c1 = c2 = 1.49445 and w = 0.729. The parameters of the SSA are set as follows: the number of producers and SD accounted for 20% and 10%, respectively, and ST = 0.8. The optimal value, mean value, and standard deviation (STD) of the objective function were calculated by running each standard test function 30 times. The standard deviation indicated the stability and robustness of the algorithm, while the mean value reflected its convergence accuracy [38].

(i) Optimisation comparison on unimodal testing functions

**Figure 8:**
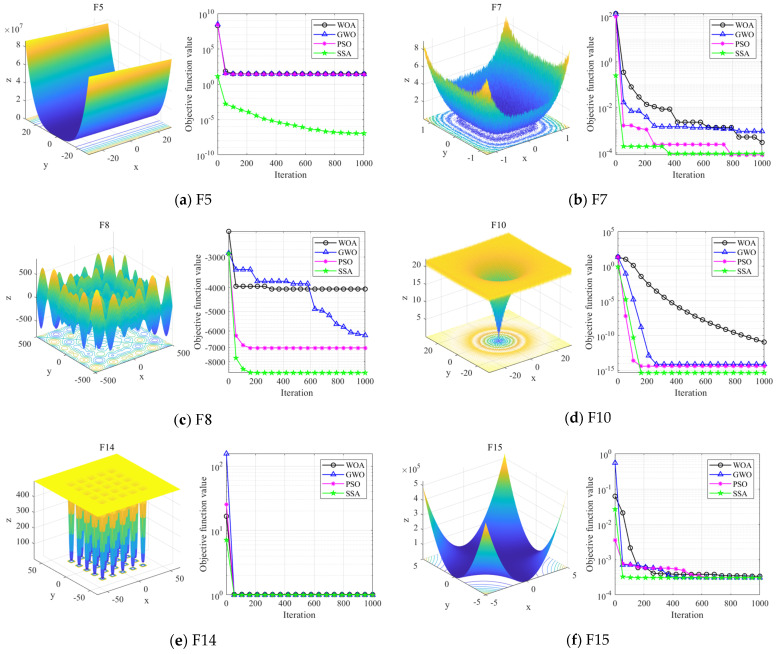
Comparison of iterative processes for different optimisation algorithms.

According to the optimal and average values in Table 2, the convergence accuracy of the SSA is significantly higher than that of the WOA, PSO, and GWO algorithms for the F5 and F7 test functions. The standard deviation (STD) data in Table 2 reveal that the SSA exhibits the smallest standard deviation for the F5 and F7 functions compared with WOA, PSO, and GWO, indicating greater stability in its results. The convergence curves for the unimodal test functions, illustrated in Figure 8a,b, further show that the SSA achieves a markedly faster convergence speed compared with WOA, GWO, and PSO.

(ii) Optimisation comparison of multimodal testing functions

In the F8 and F10 test functions, the SSA demonstrates significantly better convergence accuracy compared with WOA, PSO, and GWO, as indicated by the optimal and average values in Table 2. The standard deviation (STD) data in Table 2 show that while the standard deviation of the SSA in the F8 test function is comparable to that of GWO, it is notably smaller than that of PSO and GWO in the F10 test function, reflecting greater stability in the SSA’s results. The convergence curves for the multimodal test functions, illustrated in Figure 8c,d, show that the SSA converges to a superior value after approximately 200 iterations, achieving a significantly faster convergence speed than WOA, GWO, and PSO.

(iii) Comparison of fixed-dimensional test functions

According to the results in Table 2, the SSA effectively and quickly searches for the optimal value in the F14 and F15 test functions. For the F14 test function, the SSA demonstrates superior convergence accuracy compared with the WOA, GWO, and PSO algorithms. The STD data in Table 2 show that the standard deviation of the SSA in the F14 test function is significantly smaller than that of WOA, GWO, and PSO, and the standard deviation in F15 is zero. This indicates that the SSA achieves stable clustering around the global optimum. The convergence curves for the F14 and F15 test functions, illustrated in Figure 8e,f, illustrate that the SSA converges to a stable value after approximately 100–200 iterations, demonstrating a significantly faster convergence speed compared with WOA, GWO, and PSO.

The results indicate that the SSA has notable advantages and competitiveness compared with WOA, GWO, and PSO, as evidenced by its superior convergence speed, stability, and accuracy across unimodal, multimodal, and fixed-dimensional test functions.

## 5. Experimental Analysis

### 5.1. Experimental Equipment

**Figure 9:**
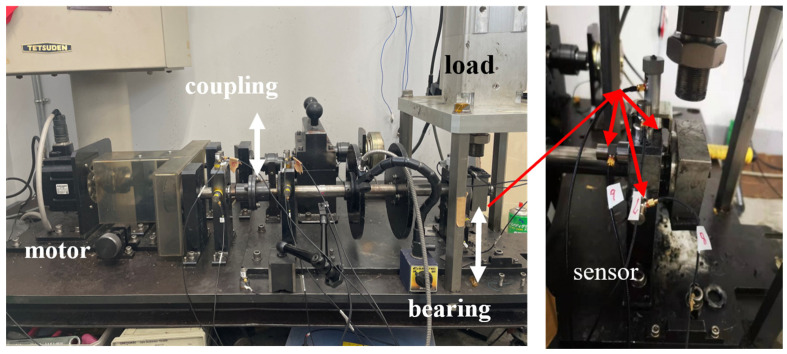
Experimental platforms.

**Figure 10:**
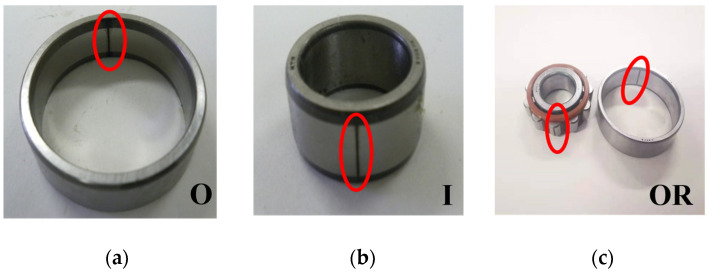
(**a**) Bearing inner fault. (**b**) Bearing outer fault. (**c**) Bearing compound fault.

This section uses data collected from the bearing failure test bench at Mie University to validate the application of the proposed SSA-VMD method for fault diagnosis in rotating machinery, as illustrated in Figure 9. The bearing type was NU204, as shown in Figure 10, with an inner ring width of 0.7 mm and depth of 0.25 mm, an outer ring width of 0.7 mm, and a depth of 0.25 mm. Nine accelerometers (PCB MA352A60) were installed in the horizontal, vertical, and axial directions on two bearing housings to capture signals at a frequency of 100 kHz for 20 s. There were nine acceleration sensors in the experimental platforms for the following reasons: (i) To ensure continuity and reliability; if one sensor failed or was damaged, other sensors could continue to collect data. (ii) To collect more detailed information for the bearing; the sensors placed at different positions simultaneously monitored vibration signals from various directions or locations of the bearing, capturing more fault-related information. Those data were applied for intelligent fault diagnosis, which is another research direction of our research group. The data from the ch-7 channel was used for the experiment in this research, which is indicated by the red arrow in Figure 9.

In this research, the working conditions considered rotational speed because the bursts (vibration components caused by bearing faults) are crucial for extracting the fault characteristic frequency from high-level noise. This noise mainly consisted of the following two sources: periodic impact components and random noise, with the bursts being proportional to the shaft speed. However, the noise level did not decrease when operating at low speeds. As a result, the low signal-to-noise ratio (SNR) made the energy of the fault characteristic frequency less prominent, which poses a significant challenge in diagnosing faults under low-speed conditions.

The bearing parameters are detailed in Table 3, and the fault characteristic frequency for the bearing operating at 500 RPM is listed in Table 4.

### 5.2. Single Fault Type Analysis

#### 5.2.1. Outer Ring Fault

The parameter combinations obtained from the SSA-VMD analysis of the outer bearing are shown in Table 5.

To further validate the effectiveness of the SSA-VMD method, the original bearing vibration signals were processed as described below to enhance the signal-to-noise ratio.

**Figure 11:**
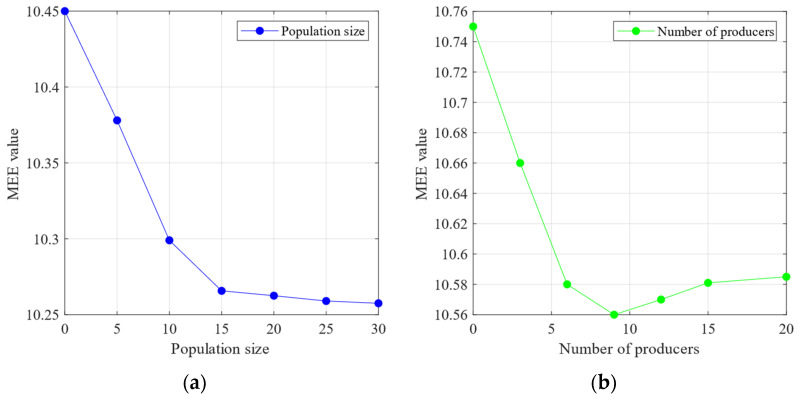
(**a**) The relationship between the population size and the mean envelope entropy. (**b**) The relationship between the number of producers and the mean envelope entropy (Outer bearing).

The sensitivity analysis of the SSA parameters—population size and number of producers—was performed using the minimum MEE introduced in Section 3 as the objective function. Figure 11a demonstrates that with the increase in the population size, the MEE reaches a minimum value of approximately 10.25. According to the theoretical analysis in Section 3, a lower MEE indicates a lower complexity in the IMF decomposition of the signal, leading to a more stable signal. In contrast, Figure 11b shows that the MEE reaches its lowest value of 10.56 when the number of producers is minimised. The MEE starts to increase with the number of producers. These findings suggest that the number of producers should not be too high, as excessive producers may introduce chaos into the optimisation process. The following experiment was conducted based on the analysis of population size and producer quantity mentioned above. This experiment set the population size to 30 and the number of producers to eight.

**Figure 12:**
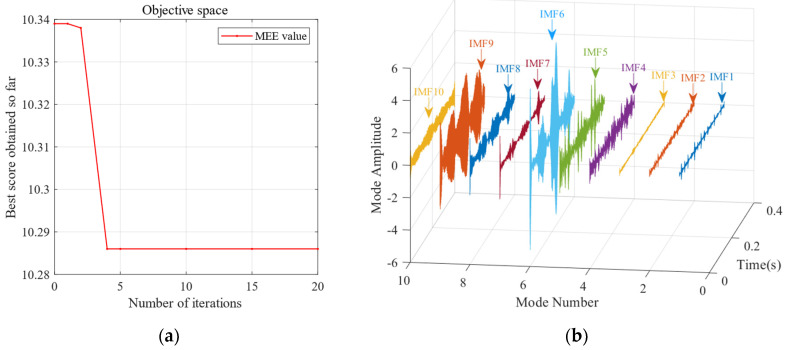
(**a**) The convergence curve of the SSA for optimising the VMD parameters of the bearing outer ring at 500 RPM. (**b**) Modes and corresponding IMF components obtained using the proposed SSA-VMD method (*k* = 10, *α* = 11,464).

**Figure 13:**
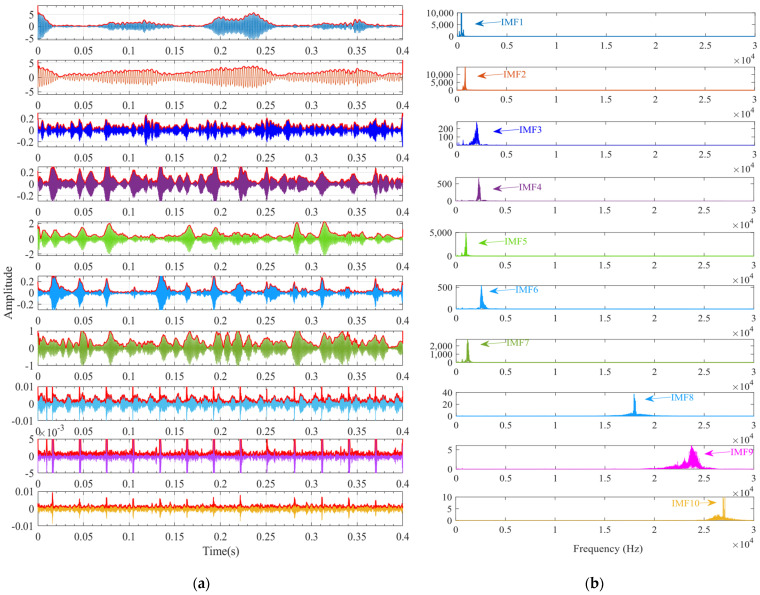
(**a**) Envelope spectra of IMF1–IMF10. (**b**) Spectrograms of IMF1–IMF10.

**Figure 14:**
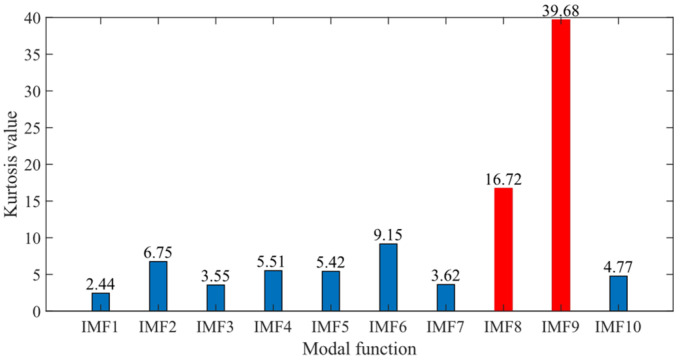
Kurtosis value of IMF1–IMF10.

**Figure 15:**
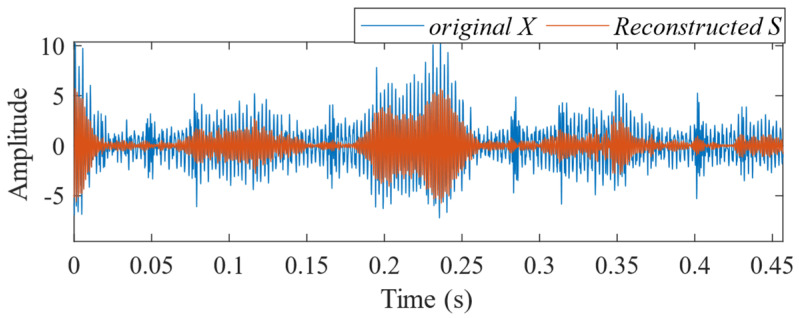
Comparison of the original fault signal and the reconstructed signal.

**Figure 16:**
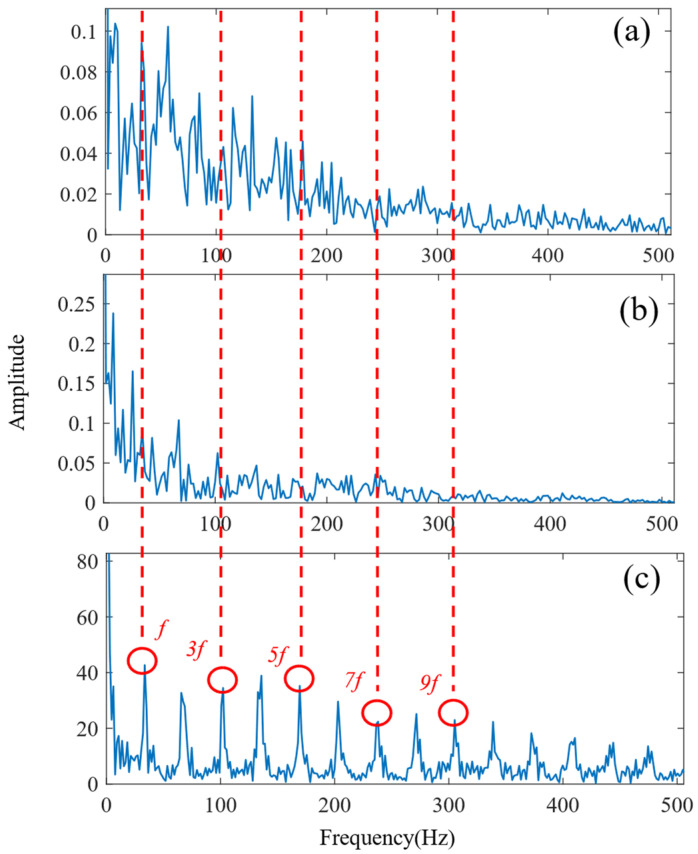
Envelope spectrum of the outer bearing (**a**) EMD method. (**b**) VMD method. (**c**) Proposed SSA-VMD method.

First, the minimum MEE proposed in Section 3 was used as the fitness function to optimise the VMD parameters *k* and α under the current working conditions through the SSA. The optimisation process is shown in Figure 12a. When the MEE reaches the minimum value within the optimisation range, the optimal decomposition parameters are determined as *k* = 10 and α = 11,464. Using these parameters, the original vibration signal was decomposed into IMF1–IMF10, as shown in Figure 12b. The envelopes of IMF1–IMF10 and their corresponding spectra are illustrated in Figure 13a,b. IMF8–IMF10 effectively separate the frequency bands. The kurtosis values of IMF1–IMF10 obtained through the decomposition are shown in Figure 14. Based on the kurtosis criterion, IMF8 and IMF9, which have higher kurtosis values, were selected to reconstruct a new signal, further isolating noise components, as shown in Figure 15. The characteristic frequency analysed from the envelope spectrum of the reconstructed signal is shown in Figure 16c. According to Table 4, the characteristic frequency of the bearing at 500 RPM is 36.5956 Hz. In Figure 16c, both the fundamental frequency and its seventh harmonic are visible, indicating a fault in the outer race of the bearing.

Figure 16a,b show the envelope spectra obtained by processing the original vibration signal using traditional EMD and VMD, respectively. However, the characteristic frequencies of the bearing faults remain obscured by noise, indicating that both EMD and VMD face challenges in effectively extracting fault signatures from low-speed bearing signals. Environmental noise and interference from other rotating components further complicate fault diagnosis, often reducing the accuracy of these traditional methods.

According to the experimental results, the SSA-VMD method proposed in this paper effectively mitigates the impact of strong background noise on low-speed bearings. It accurately and rapidly identifies weak fault characteristics of the bearing outer ring that are obscured by noise, demonstrating superior performance compared with traditional signal decomposition methods such as EMD and VMD.

#### 5.2.2. Inner Ring Fault

Table 6 shows the parameter combinations obtained by optimising the bearing inner ring using SSA-VMD.

**Figure 17:**
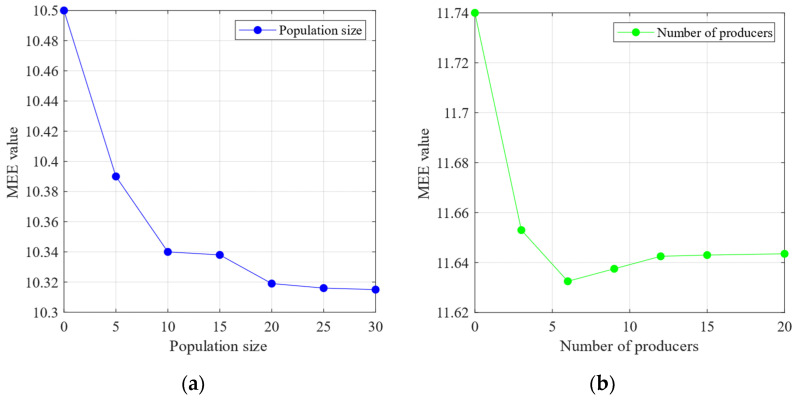
(**a**) The relationship between the population size and the mean envelope entropy. (**b**) The relationship between the number of producers and the mean envelope entropy (Inner bearing).

Figure 17 reveals that further increasing the number of producers causes the MEE to decrease again after reaching the minimum number of producers. Therefore, an excessive number of producers is avoided while establishing the SSA parameters. Instead, moderately increasing the population size can achieve the minimum MEE more effectively, enhancing the optimisation performance. Based on the above population size and producer quantity, the following experiment was conducted to assess the impact of the SSA. This experiment set the population size to 30 and the number of producers to six.

**Figure 18:**
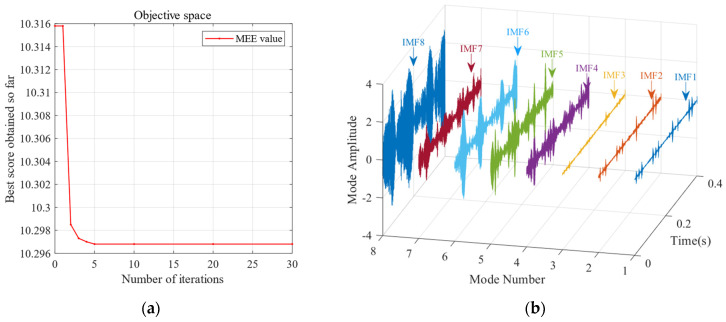
(**a**) Convergence curve of the SSA for optimising the VMD parameters of the bearing outer ring (500 RPM). (**b**) Modes and corresponding IMF components obtained using the proposed SSA-VMD method (*k* = 10, *α* = 11,464).

**Figure 19:**
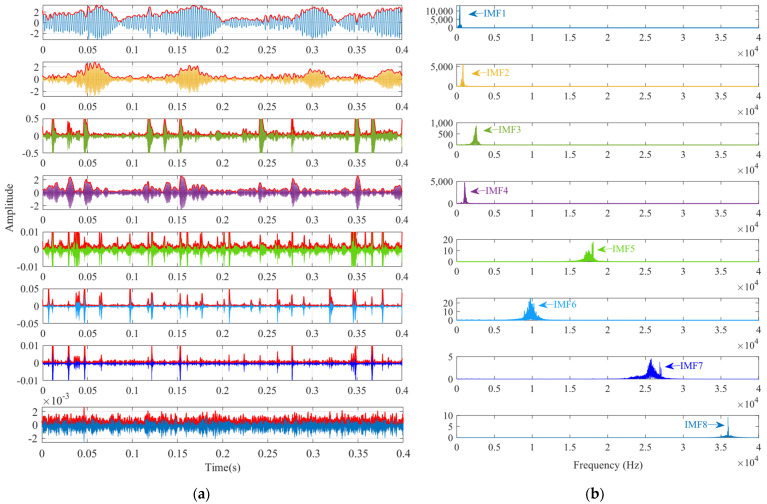
(**a**) Envelope of IMF1–IMF8. (**b**) Spectrogram of IMF1–IMF8.

**Figure 20:**
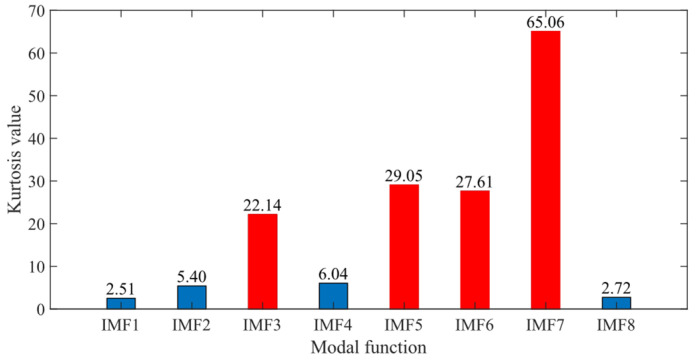
Kurtosis values of IMF1 through IMF8.

**Figure 21:**
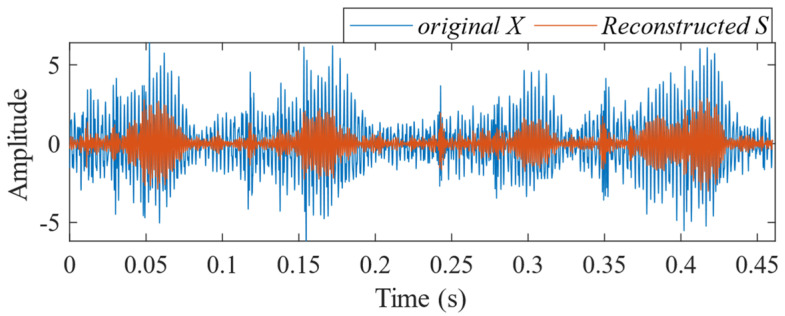
Comparison between the original fault signal and the reconstructed signal.

**Figure 22:**
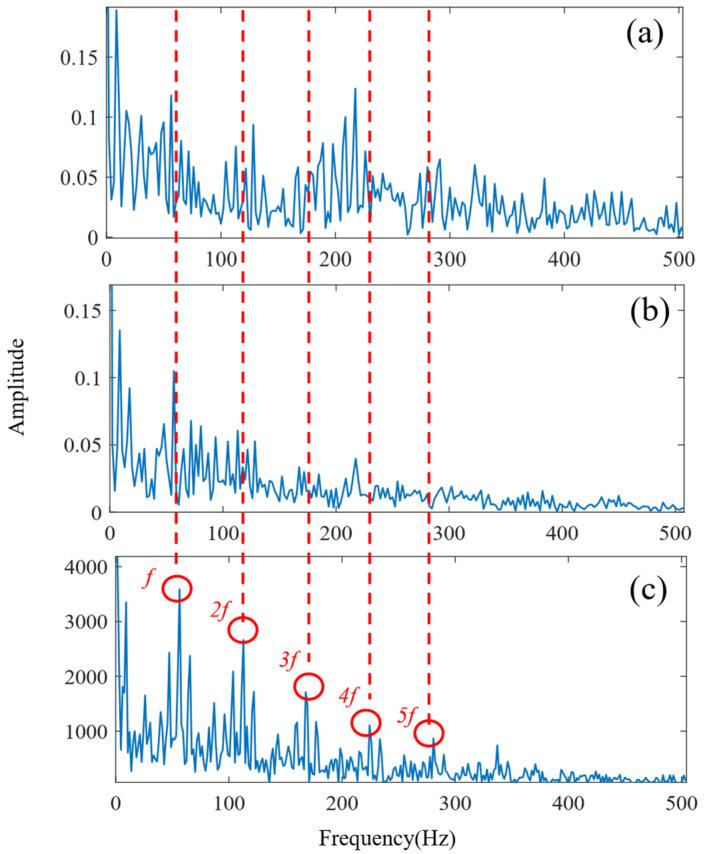
Envelope spectrum of the inner bearing (**a**) EMD method. (**b**) VMD method. (**c**) Proposed SSA-VMD method.

According to the proposed SSA-VMD method, adaptive optimisation yielded parameters *k* = 8 and *α* = 17,553, which were used to decompose the original signal into IMF1–IMF8 components via VMD. The optimisation process is shown in Figure 18a. The original vibration signal was decomposed into IMF1–IMF8, as shown in Figure 18b. The envelopes and spectra of IMF1–IMF8 are illustrated in Figure 19a and Figure 19b, respectively. IMF5–IMF8 provides effective frequency band separation. The optimised curves are illustrated in Figure 19a. The kurtosis values for the IMF components were calculated, as illustrated in Figure 20. According to the kurtosis criterion, IMF3 and IMF5–IMF7 were selected for signal reconstruction. The reconstructed time-domain signal, illustrated in Figure 21, demonstrates a reduction in environmental noise interference compared with the original vibration signal. In the envelope spectra illustrated in Figure 22a,b, the EMD and VMD methods exhibit a blurred second harmonic. In contrast, the envelope spectrum obtained using the SSA-VMD method reveals a distinct fifth harmonic relationship, as illustrated in Figure 20.

Figure 21 shows the time-domain signal of an inner race fault in the bearing, where periodic impact features are faintly discernible. However, environmental noise and interference from other components diminish the energy of these fault characteristics. Consequently, the envelope spectra generated by EMD and VMD, shown in Figure 22a,b, make it difficult to identify the bearing fault characteristic frequencies and their harmonics directly. The experimental results demonstrate that SSA-VMD effectively minimises the impact of modulating frequency components, such as environmental noise, on vibration signals. This enables the extraction of more accurate fault characteristics, which is beneficial for high-precision diagnostics.

### 5.3. Compound Fault

In this section, the bearing fault analysed is a compound fault, involving both the outer race and the roller. This type of fault is common, especially in long-term operations or under complex working conditions, where the impact of multiple faults must be comprehensively considered. The choice of 300 RPM is due to the fact that, at this lower speed, the bearing fault signals are weaker and noise interference is more pronounced compared with 500 RPM. Furthermore, the effectiveness of the SSA-VMD method can be further demonstrated by testing different faults and rotational speeds. This not only broadens the application scope of fault diagnosis technology in industrial environments but also enhances its reliability under varying operational conditions.

The parameter combinations obtained for optimising the bearing compound fault using SSA-VMD are shown in Table 7.

This section evaluates the effectiveness of the SSA-VMD method by selecting a composite fault type involving both the outer ring and rolling elements of rolling bearings. The damage to both components can interact, causing mixed vibration signal characteristics that make individual faults difficult to distinguish. Thus, the composite fault characteristics arise from the interaction of multiple faults. Consequently, further analysis of the impact response of rolling bearings under various composite fault conditions becomes essential.

**Figure 23:**
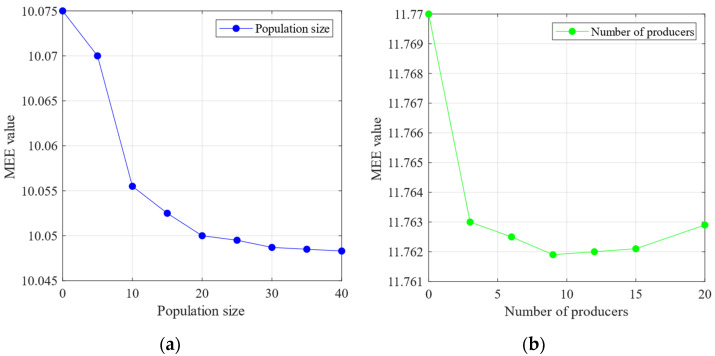
(**a**) The relationship between the population size and the mean envelope entropy. (**b**) The relationship between the number of producers and the mean envelope entropy (Compound fault).

Figure 23 reveals that with the increase in the population size, the MEE reaches a relatively low value within a certain range. In contrast, when the number of producers exceeds eight, further increases cause the MEE to rise, leading to a decline in optimisation performance. Therefore, a population size of 30–40 is used while avoiding setting the number of producers too high.

Considering the composite fault state of the outer ring and rolling elements amidst multiple interference factors, the overall signal from the bearing displays only weak fault characteristics. The impact characteristic frequencies of the bearing are obscured by harmonic and noise interference, with the fault signal being diluted by the noise. Consequently, the fault amplitudes of both the outer ring and rolling elements are relatively small.

**Figure 24:**
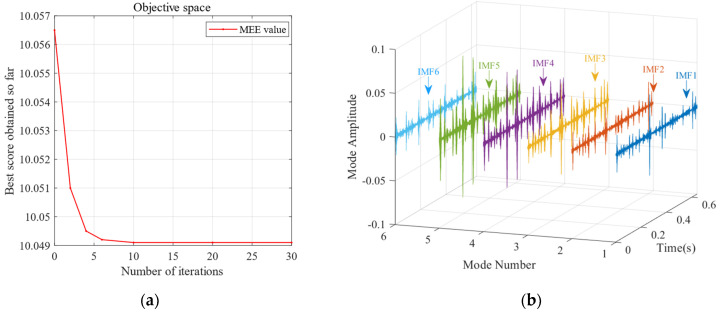
(**a**) The SSA convergence curve for optimising the VMD parameters of the bearing outer ring and rolling element (500 RPM). (**b**) Modes and corresponding IMF components obtained using the proposed SSA-VMD method (*k* = 6, *α* = 49,768).

**Figure 25:**
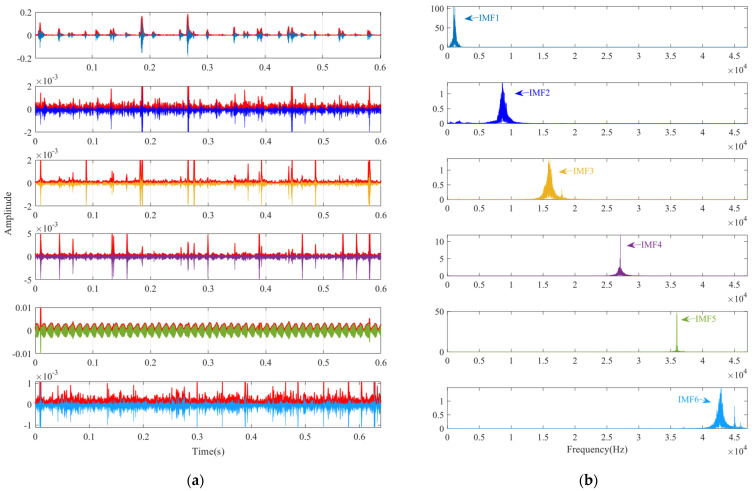
(**a**) Envelope of IMF1–IMF6. (**b**) Spectrogram of IMF1–IMF6.

**Figure 26:**
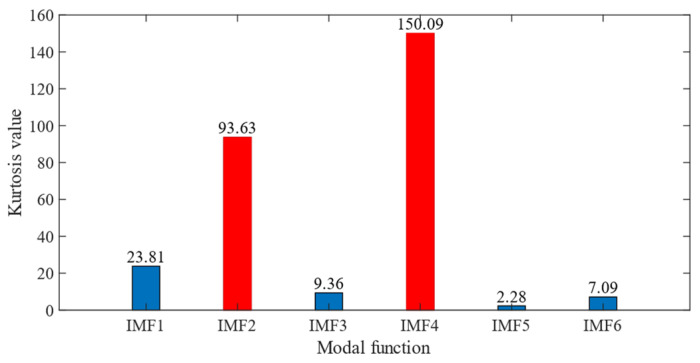
Kurtosis values of IMF1 through IMF6.

**Figure 27:**
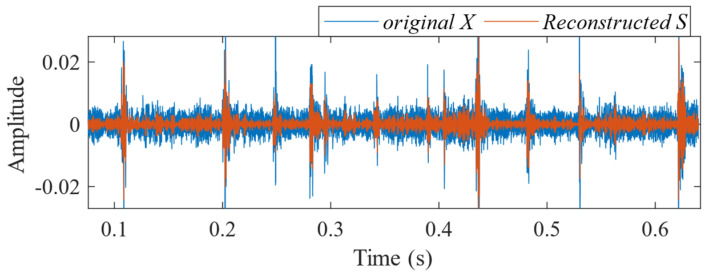
Comparison between the original fault signal and the reconstructed signal.

According to the SSA-VMD method, the optimised parameters were *k* = 6 and *α* = 49,768, with the resulting optimisation curve illustrated in Figure 24a. The time-domain signals of IMF1–IMF6 are illustrated in Figure 24b. Figure 25a,b illustrate the envelopes and spectra of IMF1–IMF6, demonstrating that the frequency bands are effectively separated. The kurtosis values of IMF1–IMF10 obtained through the decomposition are shown in Figure 26. According to the kurtosis criterion, IMF2 and IMF4 were selected for signal reconstruction as shown in Figure 27. 

**Figure 28:**
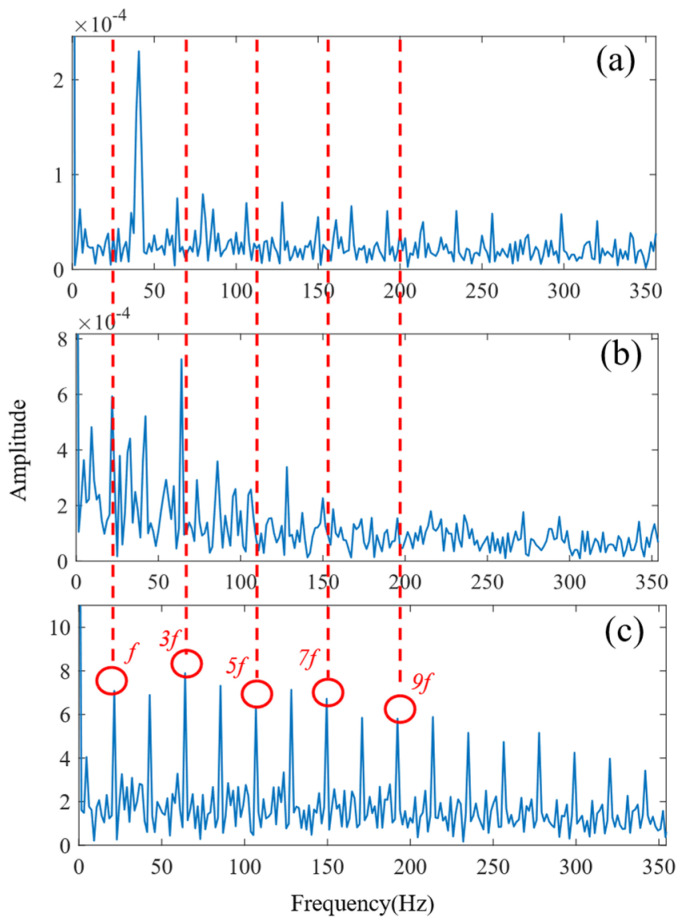
Envelope spectrum of the compound fault (**a**) EMD method. (**b**) VMD method. (**c**) Proposed SSA-VMD.

As shown in Figure 28a,b, under complex coupling conditions, the optimal envelope spectrum derived from EMD fails to disclose the fault characteristic frequencies because of noise interference. Although the optimal envelope spectrum obtained using VMD does reveal the third harmonic, other harmonic components remain significantly obscured by noise, which may result in misdiagnosis. In contrast, Figure 28c illustrates the fundamental frequency of the fault characteristics as well as its seventh- and higher-order harmonics, indicating the presence of a composite fault between the outer ring and the rolling elements of the bearing.

## 6. Conclusions

A method called SSA-VMD, which combines the SSA with parameter-adaptive VMD, is proposed for analysing vibration signals in low-speed bearing fault diagnosis. This approach effectively addresses the challenges associated with traditional VMD, such as determining the number of modes and the penalty factor, by adaptively optimising parameters using the SSA, and thus offers improved robustness and convergence. The main conclusions are as follows:

(i) The minimum mean envelope entropy is used as the fitness function. This enables the effective capture of weak signal information under low-speed conditions and the establishment of a robust link between the raw signal, VMD, and the SSA.
(ii) The incorporation of the kurtosis criterion further enhances the method by providing a strategy for selecting the optimal IMFs for signal reconstruction, thereby reducing noise interference.
(iii) The proposed method can be applied to both simulated signals and experimental signals of bearings with outer ring faults, inner ring faults, and compound faults.

The results demonstrate that SSA-VMD is an effective and adaptable approach, capable of accurately identifying fault information and successfully diagnosing faults in low-speed bearings. Compared with other commonly used techniques, the SSA-VMD method more accurately extracts low-speed bearing fault features, highlighting its strong adaptability and effectiveness in fault diagnosis.

In the future, the SSA-VMD method will be explored in greater depth. Our research will focus on achieving higher precision in diagnosing low-speed bearing faults, particularly at speeds below 100 rpm.

## Figures and Tables

**Table 1:** The test functions.

Function	Range	F_min_	Dim	Type
$F_{5}(x)=\sum_{i=1}^{n}x_{i}^{2}$	*x* ∈ [−30, 30]	0	30	unimodal
$F_{7}(x)=\sum_{i=1}^{n}ix_{i}^{4}+random\left[0,1\right]$	*x* ∈ [−1.28, 1.28]	0	30	
$F_{8}(x)=\sum_{i=1}^{n}-x_{i}\sin(\sqrt{\left|x_{i}\right|})$	*x* ∈ [−500, 500]	−418.9829n	30	multimodal
$F_{9}(x)=-20\exp\left(-0.2\sqrt{\frac{1}{n}}\sum_{i=1}^{n}x_{i}^{2}\right)-\exp\left(\frac{1}{n}\sum_{i=1}^{n}\cos(2\pi x_{i}\right)+20+e$	*x* ∈ [−32, 32]	0	30	
$F_{14}(x)=\left[2+{\left(x_{1}-7\right)}^{2}+2{\left(x_{2}-7\right)}^{2}\right]\left[1-\left|\frac{\sin\left[\pi \left(x_{1}-2\right)\right]\sin\left[\pi (x_{2}-2)\right]}{\pi^{2}(x_{1}-2)(x_{2}-2)}\right|\right]$	*x* ∈ [0, 14]	0	2	fixed dimensional
$F_{15}(x)=-{\left(\left|e^{\left|100-\sqrt{x_{1}^{2}+x_{2}^{2}/}\pi \right|}\sin(x_{1})\sin(x_{2})\right|+1\right)}^{-0.1}$	*x* ∈ [−10, 10]	−1.0	2	

**Table 2:** Results of the test functions.

Function	Algorithm	Best	Average	STD
F5	WOA	26.8864	27.86558	0.763626
	GWO	25.1280	26.0690	0.7228
	PSO	8.1702	46.5122	35.9017
	SSA	$9.9463\times 10^{-10}$	$9.2495\times 10^{-7}$	$1.9811\times 10^{-6}$
F7	WOA	$2.087\times 10^{-5}$	0.001425	0.001149
	GWO	$3.1934\times 10^{-5}$	$2.3525\times 10^{-4}$	$1.3641\times 10^{-4}$
	PSO	$3.2\times 10^{-3}$	$7\times 10^{-3}$	$2.7\times 10^{-3}$
	SSA	$9.3993\times 10^{-6}$	$1.3458\times 10^{-4}$	$1.7597\times 10^{-4}$
F8	WOA	−7056.7	−5080.76	695.7968
	GWO	−7570.5	−6347.9	615.6736
	PSO	−8700.4	−6960.8	838.1568
	SSA	−9013.0	−7726.67	698.7294
F10	WOA	$8.88\times 10^{-16}$	7.4043	9.897572
	GWO	$7.9936\times 10^{-15}$	$1.0125\times 10^{-14}$	$2.5721\times 10^{-15}$
	PSO	$7.1942\times 10^{-14}$	0.3429	0.6375
	SSA	$8.8818\times 10^{-16}$	$8.8818\times 10^{-16}$	0.0
F14	WOA	0.9980038	2.111973	2.498594
	GWO	$3.7946\times 10^{-6}$	1.7593	0.5920
	PSO	$-8.5487\times 10^{-14}$	1.9333	0.3651
	SSA	$1.7097\times 10^{-13}$	$3.9574\times 10^{-6}$	$1.3466\times 10^{-9}$
F15	WOA	0.0003076	0.000572	0.000324
	GWO	−1.0	−0.0336	0.1825
	PSO	−1.0	−0.5732	0.3936
	SSA	−1.0	−1.0	0.0

**Table 3:** Parameters of the tested bearing.

Parameter	Value
Bearing specs	NU204
Contact angle (rad) Defect on outer (mm)	0 0.7 × 0.25 (width × depth)
Defect on inner (mm)	0.7 × 0.25 (width × depth)
Defect on compound outer (mm)	0.3 × 0.05 (width × depth)
Defect on compound roller (mm)	0.3 × 0.05 (width × depth)

**Table 4:** Fault feature frequency of the bearing.

Speed	*f_o_*	*f_i_*
500 RPM	36.5956 Hz	55.0711 Hz

**Table 5:** Optimisation parameters of the bearing outer.

Speed	*k*	*α*
500 RPM	10	11,464

**Table 6:** Optimisation parameters of the inner bearing.

Speed	*k*	*α*
500 RPM	8	17,553

**Table 7:** Optimisation parameters of the bearing compound fault.

Speed	*k*	*α*
300 RPM	6	49,768

## Data Availability

The raw/processed data cannot be shared at this time. Due to the nature of this research, participants of this study did not agree for their data to be shared publicly.
